# Effect of Athletic Training on Fatigue During Neuromuscular Electrical Stimulation

**DOI:** 10.3389/fspor.2022.894395

**Published:** 2022-06-14

**Authors:** Thomas J. Abitante, Seward B. Rutkove, Kevin R. Duda, Dava J. Newman

**Affiliations:** ^1^Harvard-MIT Health Sciences and Technology, Massachusetts Institute of Technology, Cambridge, MA, United States; ^2^The Charles Stark Draper Laboratory, Inc., Cambridge, MA, United States; ^3^Department of Neurology, Beth Israel Deaconess Medical Center and Harvard Medical School, Boston, MA, United States; ^4^MIT Media Lab, Department of Aeronautics and Astronautics, Massachusetts Institute of Technology, Cambridge, MA, United States

**Keywords:** Neuromuscular Electrical Stimulation, athletic training, fatigue, spaceflight countermeasures, endurance activities

## Abstract

The purpose of this study was to explore the effect an individual's exercise training type will have on muscle fatigability during repetitive contractions induced by Neuromuscular Electrical Stimulation (NMES). Thirty-four subjects comprising of competitive athletes and controls were recruited into three cohorts: Endurance (runners/cyclists) *n* = 13; nine male, four female; 27 ± 8 years old, Explosive (Lifters/Sprinters) *n* = 11; nine male, two female; 30 ± 7 years old, and controls *n* = 10, six male, four female, 26 ± 4 years old. Subjects were placed in a custom-made leg extension rig, and received NMES against a fixed resistance (NMES-FR), to the Vastus Medialis muscle resulting in isometric leg extensions, at a duty cycle of 1 s on/3 s rest, for 20 min. The force of the isometric contractions was recorded using a Hogan MicroFet2 dynamometer, and three separate fatigue metrics were calculated to compare the different cohorts, sports within each cohort, and gender within each cohort. For every fatigue metric, the endurance group fatigued significantly less than both the explosive and control cohorts, with no difference observed between the explosive and the controls. Within each cohort, no significant difference was observed in any fatigue metric between sport or gender, but these comparisons lacked power. The results show that only high capacity endurance activity will have any effect on reducing one's fatigability during repetitive NMES. The implications of this conclusion can aid in the development of NMES regimens for use in healthy populations, such as athletic training or astronaut musculoskeletal countermeasures, as well as clinical applications when fatigue is to be minimized.

## Introduction

Neuromuscular Electrical Stimulation (NMES) is a rehabilitation tool that uses electrical pulses to elicit involuntary contractions of skeletal muscle. It can be administered in a variety of ways, including allowing directed movement to perform exercises such as rowing or cycling (Frotzler et al., 2008), or restraining movement with a fixed resistance to create isometric contractions. NMES is commonly used to counteract the co-morbidities associated with the spinal cord injury and has been shown to maintain bone mineral density (Dudley-Javoroski et al., 2012), increase muscle cross sectional area (Neumayer et al., 1997), and increase muscle aerobic capacity (Martin et al., 1992) with repeated use. Additionally, NMES has found use in healthy populations. This includes athletes, in whom NMES can be used as a method of strength training (Maffiuletti et al., 2000, 2002) or as a method for muscle recovery (Babault et al., 2011). Recently, NMES has been presented as a potential tool for astronauts (Maffiuletti et al., 2019; Abitante et al., 2020), who experience musculoskeletal atrophy similar to that of SCI patients (Afshinnekoo et al., 2020).

NMES elicits muscle contractions in a very different pattern from that of voluntary motor contractions. Normal recruitment follows a principle where based upon need, smaller motor units, consisting of generally Type I muscle fibers (slow twitch, low force, fatigue resistant) are recruited first, progressing to larger ones containing generally Type II muscle fibers (fast twitch, high force, fast fatiguing) (Henneman et al., 1965). In contrast, NMES at sufficient strength activates all muscle fiber in the vicinity of the electrodes simultaneously (Bickel et al., 2011). This results in rapid muscle fatigue because (1) additional muscle fibers cannot be recruited as those activated by the electrode fatigue, (2) the simulation frequency is fixed, exhausting muscle (Carpentier et al., 2001) and preventing modulation as observed during voluntary contractions (Chou et al., 2008) and (3) large Type II fibers are not designed for sustained activity (Zierath and Hawley, 2004).

Numerous studies have examined how different aspects of NMES affect fatigue and what can be done to minimize it, as well as the effects on force production. This includes the electrical parameters such as the pulse shape (Gregory et al., 2007), the pulse duration and amplitude (Behringer et al., 2016), the pulse frequency (Gregory et al., 2007), and the duty cycle, (the ratio of the active stimulation period to the rest period) (Lieber and Kelly, 1993), and the electrode characteristics such as size (Alon et al., 1994), type (Pietrosimone et al., 2011), shape and distribution (Vieira et al., 2016; Laubacher et al., 2019). Additionally, repeated NMES treatments can reduce fatigue and increase force output over time through adaptation (Rabischong and Ohanna, 1992).

However, when designing NMES protocols for use in healthy people, whether to increase athletic performance or reduce the musculoskeletal atrophy experienced by astronauts, little data exists on how fiber type predominance in a given muscle could impact fatigability and force production. Muscle fiber type composition is thought to influence athletic performance in a particular sport (Costill et al., 1976; Fink et al., 1977), and the associated muscle enzyme and mitochondria levels can heavily influence an individual's endurance capacity (Foster et al., 1978; Hawley and Stepto, 2001). While numerous studies have investigated how repeated NMES treatments in healthy individuals can affect these factors to varying results (Pérez et al., 2002; Gondin et al., 2011; Sillen et al., 2013), no study has observed how these innate muscle characteristics could affect fatigue or force production during NMES. Whether athletic specialization and performance have any effect on one's reaction to NMES is still unknown. NMES has been applied to athletes for endurance (Veldman et al., 2016), strength (Maffiuletti et al., 2000, 2002), and recovery (Babault et al., 2011), and estimating the potential maximum force output or rate of fatigue can influence specialized regimen designs. Astronauts could require either long, muscle-focused regimens (Maffiuletti et al., 2019), or short, maximum force-producing regimens for bone (Abitante et al., 2020). Additionally, as a result of disuse, spinal cord injury patient's muscles will atrophy in a pattern that results in overwhelmingly Type II muscle (Martin et al., 1992). How a healthy individual with predominately Type II muscle fiber will fatigue may help infer the design of NMES regimens for SCI patients, especially those targeting bone loss where minimizing fatigue is important (Shields et al., 2006). This study serves as an initial investigation into how the characteristics of a healthy individual's muscle, inferred through exercise training type, could affect the fatigability and force production during repetitive contractions administered with NMES.

## Methods

### Subject Selection

Thirty-four subjects of varying training types volunteered to participate in this study and were classified into one of three cohorts: Endurance athletes (*n* = 13; nine male, four female; 27 ± 8 years old), who comprised distance runners and distance cyclists, Explosive athletes (*n* = 11; nine male, two female; 30 ± 7 years old), who comprised of powerlifters, Olympic weightlifters, and sprinters, and Controls (*n* = 10, six male, four female, 26 ± 4 years old), who comprised of non-specifically trained individuals. As we were unable to obtain muscle biopsies to confirm each subject's muscle characteristics, highly competitive athletes were sought out for this study in order to represent the extremes of muscle fiber type composition and muscle enzyme levels based on prior muscle biopsy studies (Costill et al., 1976; Tesch and Karlsson, 1985), where competitive endurance athletes tend to have predominately Type I muscle fiber (≈70% Type I) and competitive explosive athletes tend to have predominately Type II muscle fiber (25–55% Type I). Furthermore, while training can affect muscle fiber type compositions (hypertrophy, shifting), there is a tendency for athletes with a genetic predisposition to a certain fiber type to be attracted to and more likely to succeed in a given sport type (Astrand et al., 1970).

The experimental procedures were approved by the Institutional Review Board at the Massachusetts Institute of Technology, protocol number 1810570889. We recruited all subjects from the greater Boston area. All subjects were required to be free of any ongoing knee injury and provided written informed consent prior to participating in the study. We instructed subjects to avoid strenuous lower body exercise for at least 24 h prior. Inclusion criteria for the explosive and endurance required individuals to be active competitors within their sport, in addition to sport-specific requirements. Inclusion criteria for the controls required that the individual was not a participant in any organized sports team or club, and engaged in no more than 3 h per week of a particular exercise type. A full list of subject specialties, as well as inclusion criteria, can be found in the Appendix (Tables A1, A2).

With a tape measure, we measured each subject's femur lengths, tibia lengths, thigh cross sectional areas at the knee, and thigh cross sectional areas at the midpoint of the femur were measured using a body tape measure. We measured the subcutaneous fat thicknesses of the legs at the distal and the midpoint of the femur using calipers. Each measurement was performed 2 times and the average was recorded. We instructed subjects to ingest no food or drink for 2 h prior to testing (Selkow et al., 2011).

### Equipment

The NMES device used in this study was a custom-built, single channel, voltage-controlled device developed at the Massachusetts Institute of Technology (MIT) Human Systems Lab (HSL) in collaboration with MIT Portugal. The device comprises an Arduino microcontroller and can be controlled via MATLAB or the Arduino software and a muscle stimulation unit (MSU), which has two connections for cutaneous electrode attachment (de Melo et al., 2015). The MSU delivers a biphasic pulse with four individually customizable parameters: pulse amplitude (V), pulse frequency (Hz), and positive and negative pulse widths (μs), which when combined are referred to jointly as the pulse width or pulse duration. Additionally, the duty cycle can be customized through the Arduino software.

**Figure 1:**
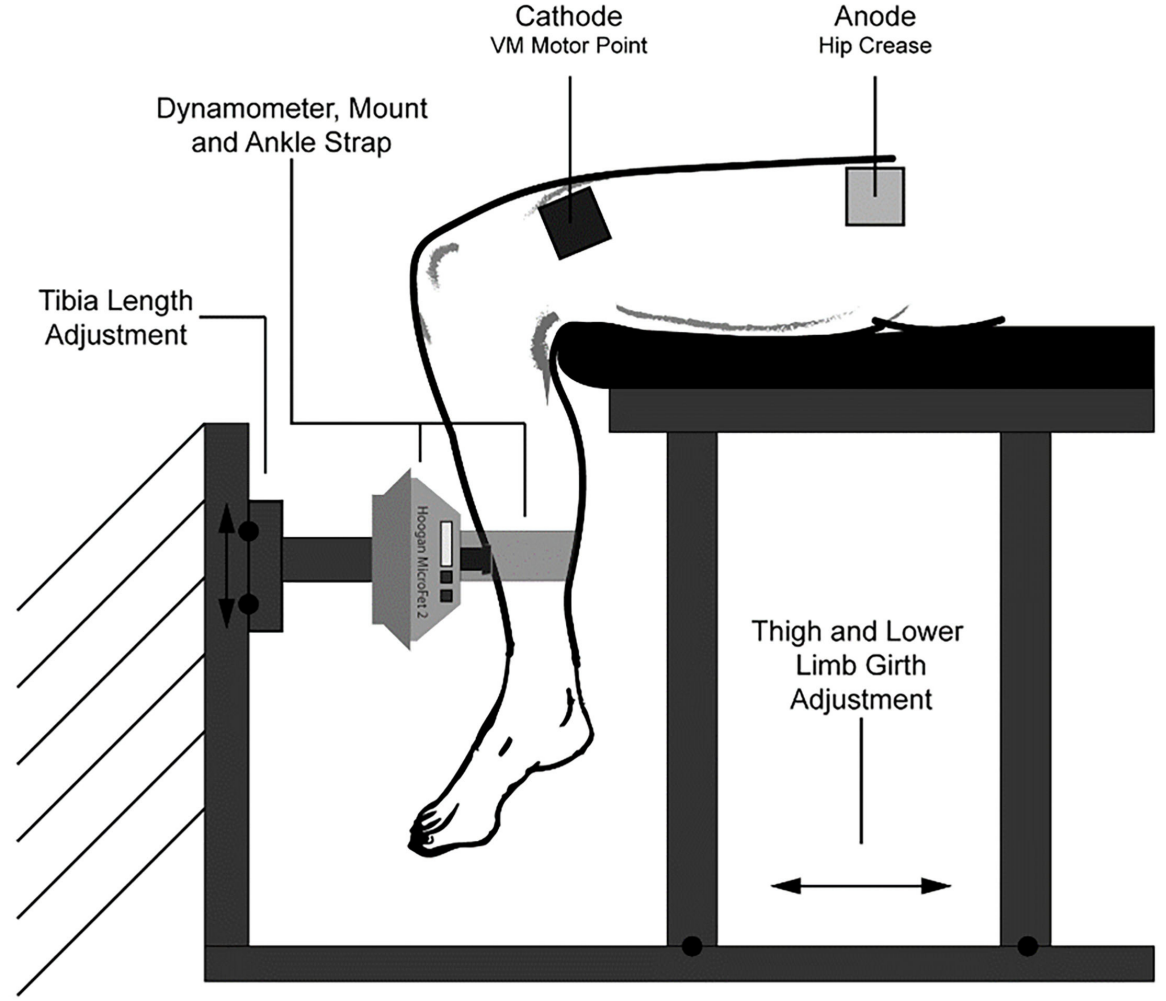
Experimental setup. Comprised of a Hoogan MicroFET 2 dynamometer that was fastened to a rigid structure, and an adjustable seat constructed from aluminum t-slot bars. The dynamometer could be adjusted vertically to account for differences in lower limb length, and the seat could be slid along a base to adjust for subject thigh length and girth. Not depicted are a back rest and hip restraint belt. This figure was originally published in the ASCEND 2020 Conference Proceedings (Abitante et al., 2020).

We constructed a knee extension dynamometer rig for this study (Figure 1) (Abitante et al., 2020). The rig was designed to prevent movement, resulting in isometric, knee extension muscle contractions. Therefore, the NMES administered in this fashion can be considered NMES against a fixed resistance (NMES-FR) The rig utilizes a Hoogan MicroFET 2 (MF2) handheld dynamometer for force measurement, which was secured with a 3D printed mount. The rig was adjusted so that when the lower shin of the subject was firmly pressing on the MF2, the knee angle was just beyond 90 degrees, measured from full extension. Having the knee angle just beyond 90 degrees, as well as a securing strap fastened around the ankle, ensured constant positive contact with the MF2, which was required at all times as knee extension force at the shin, not torque at the knee, was being recorded.

### Fatigue Test

#### Protocol

All subjects participated in the fatigue test. The fatigue test comprised of 300 NMES-FR contractions, delivered at 1 s on, 3 s off, over a 20-min time period. We chose the duty cycle of 1 second on, and 3 s off, in order to achieve a high volume of contractions and to ensure high fatigue could be observed. The NMES was delivered to the vastus medialis (VM) muscle on their dominant leg (right leg if no dominance is declared). We chose the VM due to the superficiality of the muscle, and more consistent distribution of the muscle motor end points (Botter et al., 2011) to facilitate a more consistent response between subjects. We placed two 2”x 2” square electrodes (Ultrastim X, Axelgaard) on the subject; one placed on the center of the muscle belly of the VM as observed when the subject was at full leg extension, and the other placed on the proximal thigh, below the hip crease longitudinally in line with the other. The proximal electrode placement served to maximize the inter-electrode spacing to maximize comfort and to reduce the attenuation effects the subcutaneous body fat has on NMES (Doheny et al., 2010). We then administered a familiarization protocol, which consisted of five sets of three contractions (1s on, 3s off, 10s between sets), of increasing stimulation pulse width and therefore intensity. This served to confirm optimal electrode placement and to allow the subject to become more familiar with the sensation in order to reduce involuntary, reactionary movement during the application of the fatigue test which would yield erroneous force output results. If the electrodes required adjustment more than once, we replaced them to ensure maximum adhesion. Subjects were then seated and secured in the dynamometer rig.

To account for differences in sensitivity individuals can have to NMES, we calibrated the voltage to provide an equal level of muscle activation across subjects. Normally, there is a positive linear relationship between the NMES pulse current and the percent maximum voluntary isometric contraction (MVIC) produced (Maffiuletti, 2010), however as the device used in this study is voltage controlled, the actual current delivered will vary based on variations in skin characteristics, furthering the need for a voltage calibration.

Each subject first performed two dominant leg MVICs with a 1 min rest between. Additional MVIC trials were performed if the maximum force output of the first two trials differed by more than 20%. We recorded the highest measured force value in newtons and then estimated the maximum isometric VM forces (MVIC_VM_) using the ratio of the VM's estimated physiological cross-sectional area to that of the entire quadriceps (0.255) (Akima et al., 1995). Next, we determined the NMES voltage required to elicit an isometric knee extension force measuring 20% of the MVIC_VM_ for each individual subject. The target 20% was chosen in order to maximize contractile force while avoiding excessive discomfort, based on previous NMES studies(Mayr et al., 1999; Vromans and Faghri, 2017). We administered multiple series of 3 NMES-FR contractions to each subject with the following pulse parameters: 60 Hz frequency, 400 μs pulse width at 1 s on, 3 s off. A pulse frequency of 60Hz was chosen to ensure tetanic contractions, as frequencies between 50 and 100Hz are usually required (Maffiuletti, 2010). We adjusted the voltage for each series until 20% of the individual's MVIC_VM_ was achieved. Rest between each attempt was 2 min to avoid fatigue, and no more than eight attempts were made. Subjects who exceeded the 20% target and subjects who did not reach 20% but could achieve tetanic contractions were included.

After a 5 min rest, the fatigue test began using the determined voltage and the pulse width and frequency used in calibration. We instructed subjects to avoid any movement during the 20-min duration of the test and we recorded the time of any moment when the subject made any involuntary or voluntary movement not directly related to the NMES-FR contraction which would produce erroneous data point, including readjusting position or a jerking motion. The MF2 recorded data via a Bluetooth connection, and in the event of a disconnection, the test would continue, unless the disconnection occurred within the first 2 min, or lasted more than 2 min. In the event of either condition, the test was suspended and restarted after a 20-min rest period.

####  Data Processing

The MF2 can only record 2 min at a time. This resulted in 10 data files, which we stitched together. The reported force output was in newtons, which we normalized against each subject's MVIC_VM_. We then used MATLAB to process that data to acquire each individual contraction's normalized force output. First, we used a peak finder to identify every NMES-FR contraction, with any erroneous peaks found by the software or as noted due to subject movement removed. We then applied an envelope to highlight each subject's “resting” force resultant from the active pressing of the shin against the MF2 in the time between contractions. Each contraction was then calculated using the peak and the value of the envelope at the peak's timestamp. To account for the inherent noise present with this method of force measurement (external vs. torque), we smoothed the data using a moving mean with a window of 20. Last, we removed the first five data points from all subjects to account for subjects initial adjustment to the NMES pulses. For each subject, the following fatigue metrics were determined:

- The Endurance Index (EI) at the 10 min (EI10) and the 20 min (EI20) timestamps, using the average of five contractions around each timestamp.
- The Normalized Force Area (NA) (Buckmire et al., 2018), accounts for changes in the contractile pattern over the course of the 1 second of active NMES.
- Time to Fatigue to 90% of the initial force (TF90) and the Time to Fatigue to 75% of the initial force (TF75), to account for differences in the overall fatigue profile during the trial. The Time to Fatigue (TF) calculations used a moving mean of 30 contractions.

**Figure 2:**
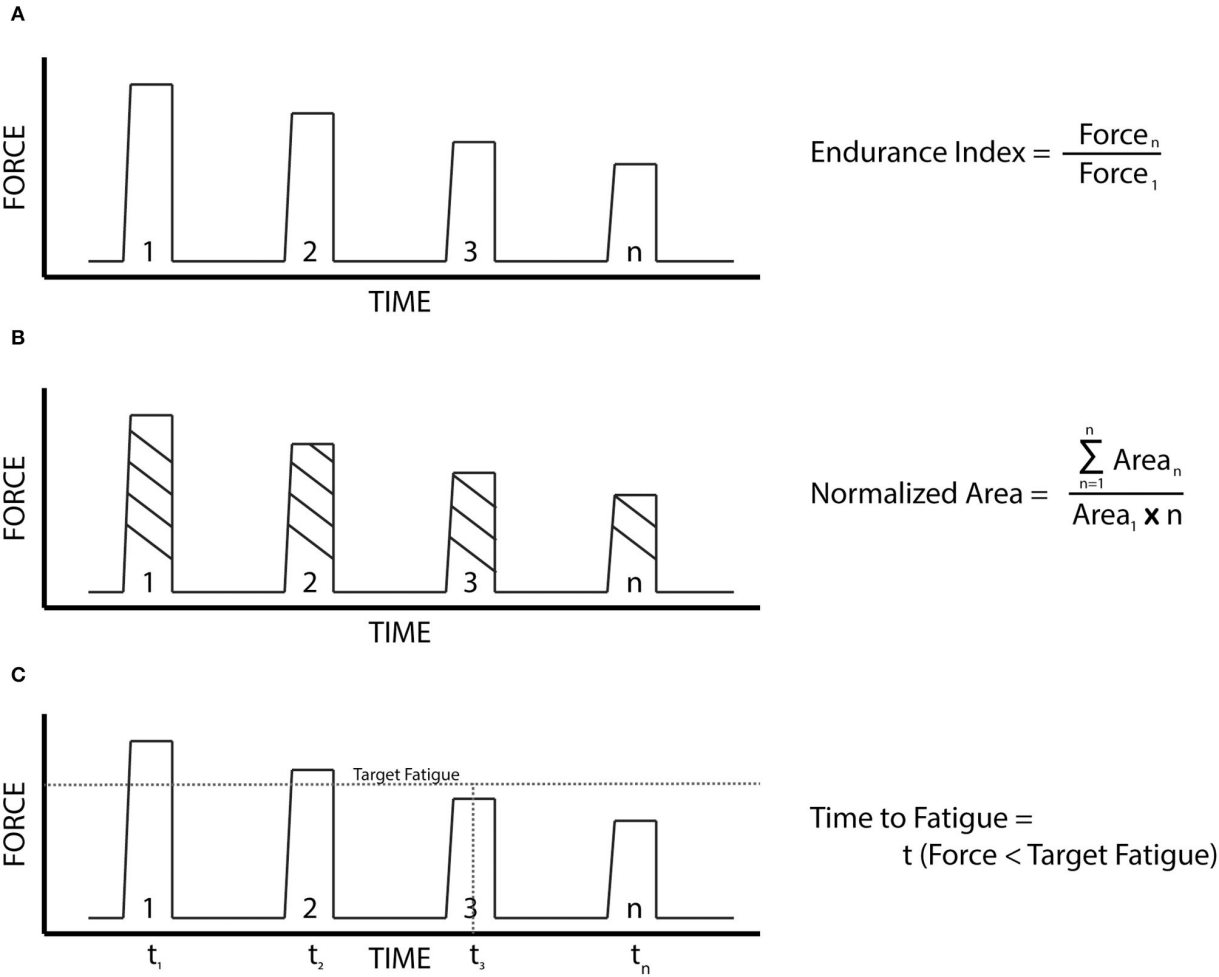
The fatigue metrics. **(A)** Endurance index: the ratio of the force output at a designated time stamp to that of the initial force output. **(B)** Normalized force area: The ratio of the total area under the entire force output profile (summation of the area of each contraction times the duration of each contraction), to that of a force output profile if each contraction was identical to the first. **(C)** Time to fatigue: the time for the force of a contraction to fall below a designated target.

These metrics were used to compare the differences in fatigue between the three cohorts, as well as each cohort, was broken down by specific sport and gender. A pictorial representation of the three fatigue metrics is displayed in Figure 2.

### Statistical Analysis

We used the Graphpad Prism version 8.0 software for all statistical analyses. All data sets were tested for normality with a Shapiro–Wilk Test. Two-way repeated measures ANOVA was performed for the EI and TF metrics, and an ordinary one-way ANOVA was performed for the NA metric. The resultant *p* values for each metric represent the *Post-hoc* test comparing the individual groups to one another. Statistical Significance was denoted as *p* < 0.05. All results are displayed as mean ± standard deviation.

## Results

### MVIC and NMES Calibration

The percent of the subjects MVIC_VM_ achieved by the NMES-FR was 21.41 ± 5.12, 22.18 ± 3.99, and 20.16 ± 4.56 for the endurance, explosive, and control groups, respectively. As the NMES device used in this study was a voltage controlled, the actual current delivered to the motor end plates could vary due to differences in skin characteristics. Therefore, we compared the pulse amplitudes between cohorts to determine if there could be any influence on the results. The amplitudes of the NMES pulses were 32.62 ± 5.36, 29.72 ± 5.93, and 34.98 ± 5.06 volts for the endurance, explosive, and control groups, respectively. There was no statistically significant difference between any group in either the MVIC_VM_ or the amplitude of the NMES pulses.

**Figure 3:**
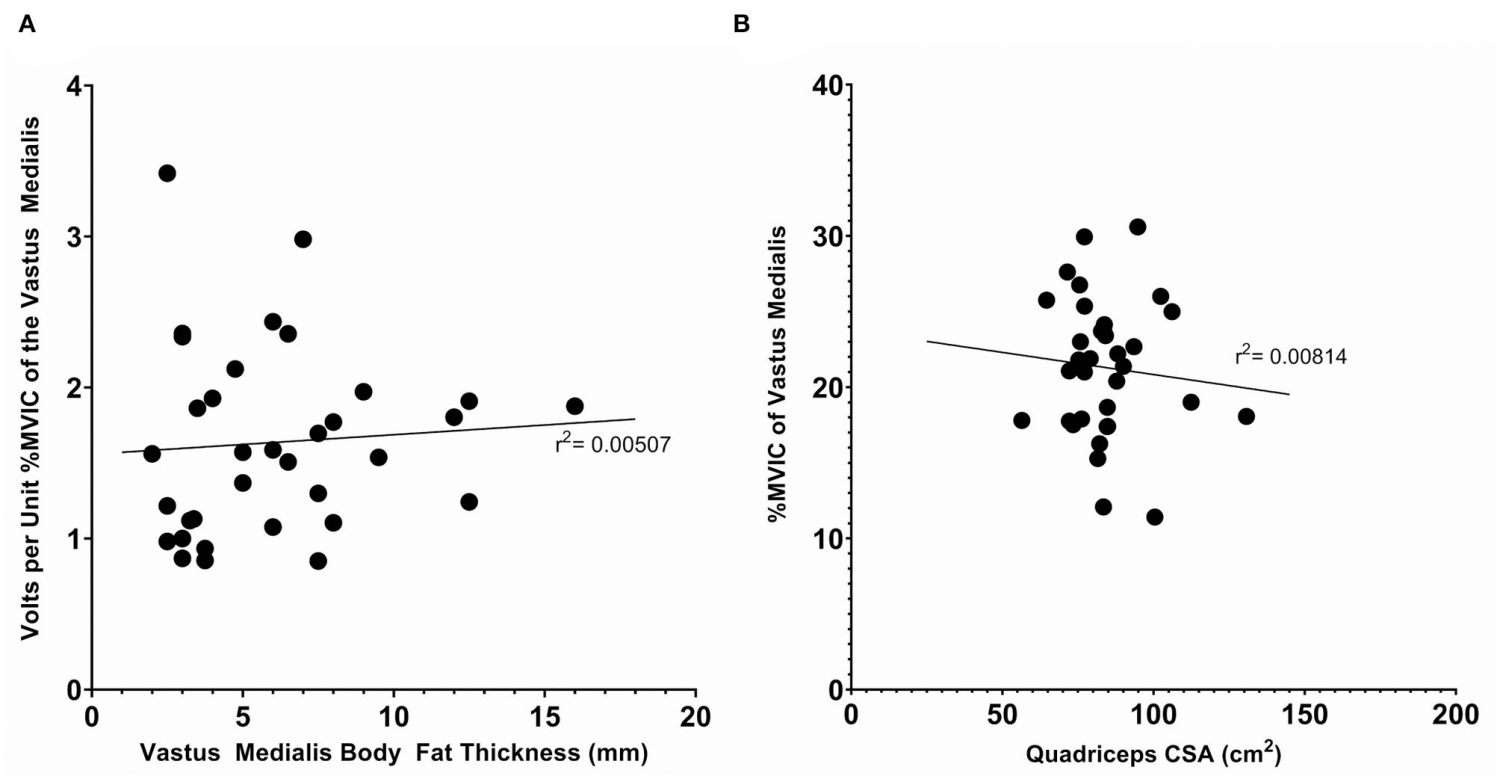
Anthropometric measurements vs. calibration data for the complete subject set (*n* = 34). **(A)** The subcutaneous body fat thickness, as measured with skinfold calipers, *vs*. the volts per unit %MVIC of the Vastus Medialis. **(B)** The quadriceps cross sectional area, as estimated with the mid-thigh circumference and distal thigh, subcutaneous body fat thickness, as measured with skinfold calipers, vs. the %MVIC of the Vastus Medialis achieved. Displayed r^2^ values denote the coefficient of determination of a linear regression.

To determine if anthropometric characteristics potentially influenced the calibration, we performed linear regressions to analyze the relationship between anthropometric and calibration data. We analyzed the relationship between the thickness of the distal thigh subcutaneous fat and volts per unit MVIC_VM_ and the relationship between the estimated quadriceps cross sectional area (Housh et al., 1995) and the total percent MVIC_VM_ achieved. These relationships are displayed in Figure 3. The r^2^ were 0.00507 and 0.00814 for each, respectively, indicating no correlation in either case.

### Data Collection

The total number of NMES-FR contraction data points recorded were 240 ± 13 (*n* = 12), 242 ± 11 (*n* = 11), and 238 ± 11 (*n* = 10) for the endurance, explosive, and control group respectively. One endurance subject's trial was removed from the study, due to an inability of the subject to tolerate the NMES-FR contractions, resulting in unfilterable noise. During the application of the fatigue test, two technical issues reduced the total number of total NMES-FR contractions recorded from the expected 300. First, the NMES device used in this study was not designed to perform repetitive pulses at a set cadence for an extended duration. Therefore, a periodic misfire occurred where only the positive or negative half pulse would be delivered, resulting in non-tetanic, or reduced force contractions. The total number of misfires varied per trial but as these misfires occurred periodically between 10 and 20 successful NMES-FR contractions, they were removed from the data set without altering any prevailing fatigue trend.

Second, the MF2 communicated with the computer software via Bluetooth, and the connection was occasionally lost, potentially due to interference from other equipment in the MIT HSL. This occurred with 1 endurance and 8 of the controls. In only the case of the endurance subject did the disconnection occur over the timestamp where an EI was measured. This metric was achieved using linear interpolation, and was included in the study as this subject was the least fatiguing of all 34 subjects and the estimated EI was deemed representative of the observed prevailing fatigue profile. The disconnections did not affect the NA calculations, as the total number of “no fatigue contractions” was correspondingly reduced. For the TF metrics, a linear interpolation was used to fill in the missing data points. A complete list of the subjects who had disconnections, and the corresponding times, is listed in the Appendix (Table A3).

### Fatigue Test

#### Cohort

**Figure 4:**
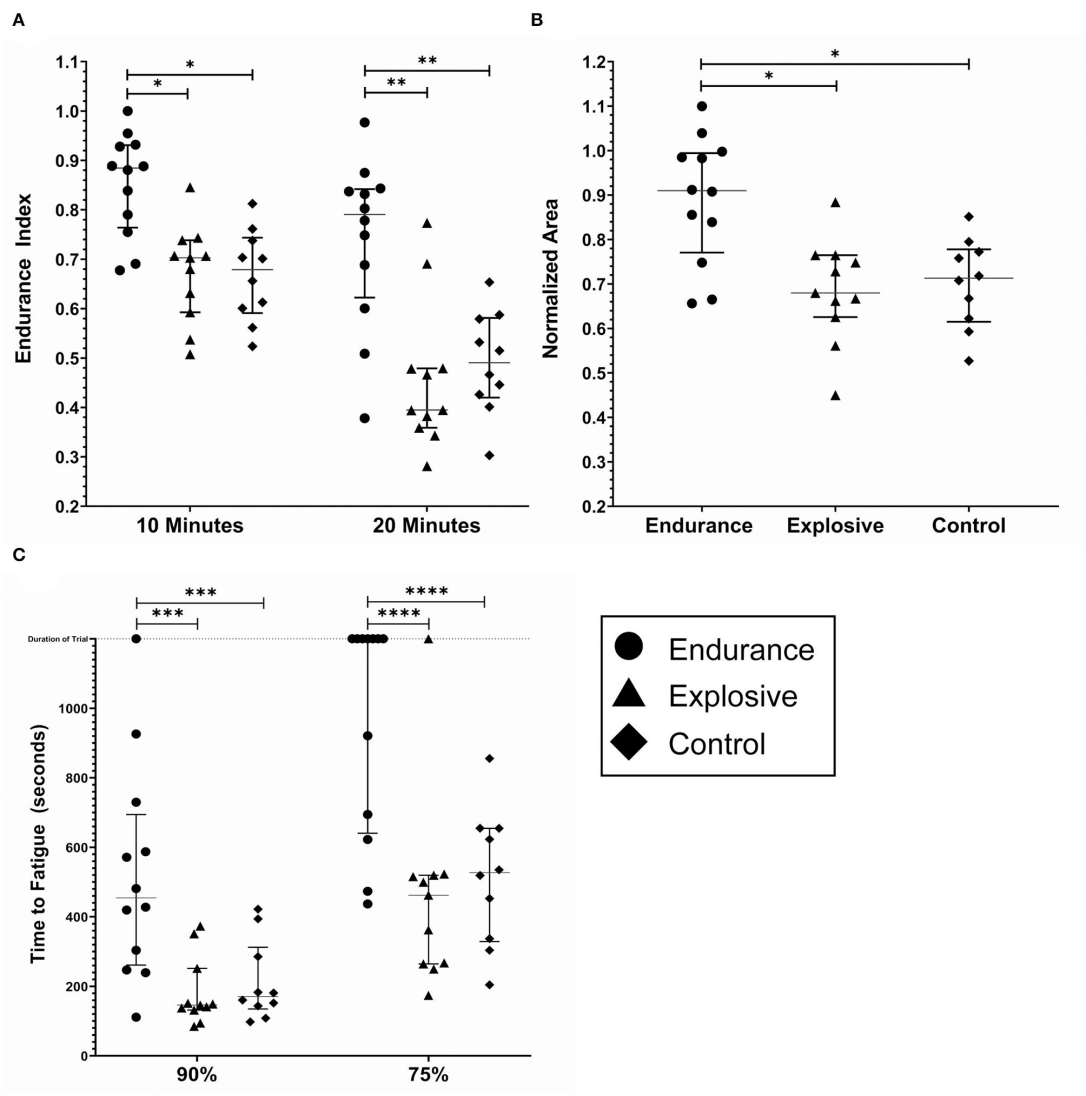
Fatigue metrics by cohort; endurance (*n* = 12), explosive (*n* = 11) and control (*n* = 10). **(A)** The endurance index as measured at the 10 and 20 min timestamp. **(B)** The Normalized Area. Some subjects had calculated values above 1.0, due to the processing method. **(C)** The time to Fatigue to 90% and 75% of the initial force output. The duration of the trail was 1,200 s and is noted by the dashed horizontal line. The lines within each data set denote the median and the interquartile range. *(*p* < *0.005)* **(*p* < 0.0001) ***(p < 0.05) ****(p < 0.0005).

Individual results for the EI and NA metrics are shown in Figures 4A,B, respectively. All data sets for the EI and NA pass a Shapiro–Wilk Normality Test. The grouped EI10s were 0.86 ± 0.1, 0.67 ± 0.1, and 0.67 ± 0.1 for the endurance, explosive, and controls groups, respectively. The endurance group's E10 value was significantly greater than both the explosive and control groups (*p* < 0.005). There was no significant difference between the explosive and the control group. The grouped E20s were 0.74± 0.17, 0.48 ± 0.12, and 0.49 ± 0.1. For E20, the endurance groups results were significantly greater than both the explosive and control groups (*p* < 0.0001). While there was no significant difference between the explosive and control groups, the control group was trending to have lower fatigue. Also observed were two low fatiguing outliers in the explosive group.

Similar results were found for the NA metric. Due to the processing method, the results of the NA calculations were inflated. The grouped NAs were 0.89± 0.14, 0.69 ± 0.12, and 0.70 ± 0.1 for the endurance, explosive, and controls groups, respectively. The endurance group's NA was significantly greater than both the explosive and control groups (*p* < 0.005), with no significant difference between the explosive and control groups.

Individual results for the TF metric are shown in Figure 4C. Seven endurance and one explosive subject never fatigued below 75% of the initial values, and one endurance subject never fatigued below 90% of the initial value. For these cases, the maximum time, 1,200 s, was included as their data point as we were unable to predict when, if at all, the subjects would reach 90% or 75%. The grouped TF90s were 520 ± 311, 183 ± 98, and 213 ± 115 s and the grouped TF75s were 962 ± 316, 458 ± 278, and 514 ± 196 s for the endurance, explosive, and control groups, respectively. In both cases, the endurance group's Time to Fatigue was significantly greater than both the explosive and control groups (*p* < 0.05, *p* < 0.0005), with no significant difference between the explosive and control groups. One endurance subject never fatigued below 90%, and seven never fatigued below 75%. There was one outlier observed in the explosive group who never fatigued below 75%. Due to the ceiling effect, the Endurance group's T75 data set did not pass a Shapiro–Wilk Normality Test, however, an extremely high degree of significance was still observed.

There was no observable trend in the fatigue profile in any cohort. In all three cohorts, linear fatigue profiles were present. Each cohort also had subjects who had fatigue profiles that appeared to have an initial linear rapid fatigue during the initial minutes, followed by shallow linear fatigue. Few subjects also had fatigue profiles that appeared exponential. However, due to the inherent noise observed as well as the tendency for some subjects to continue to have involuntary reactionary movements in the beginning, (despite the removal of the first 5 points to account for this), no formal analysis could be performed.

#### Sport

**Figure 5:**
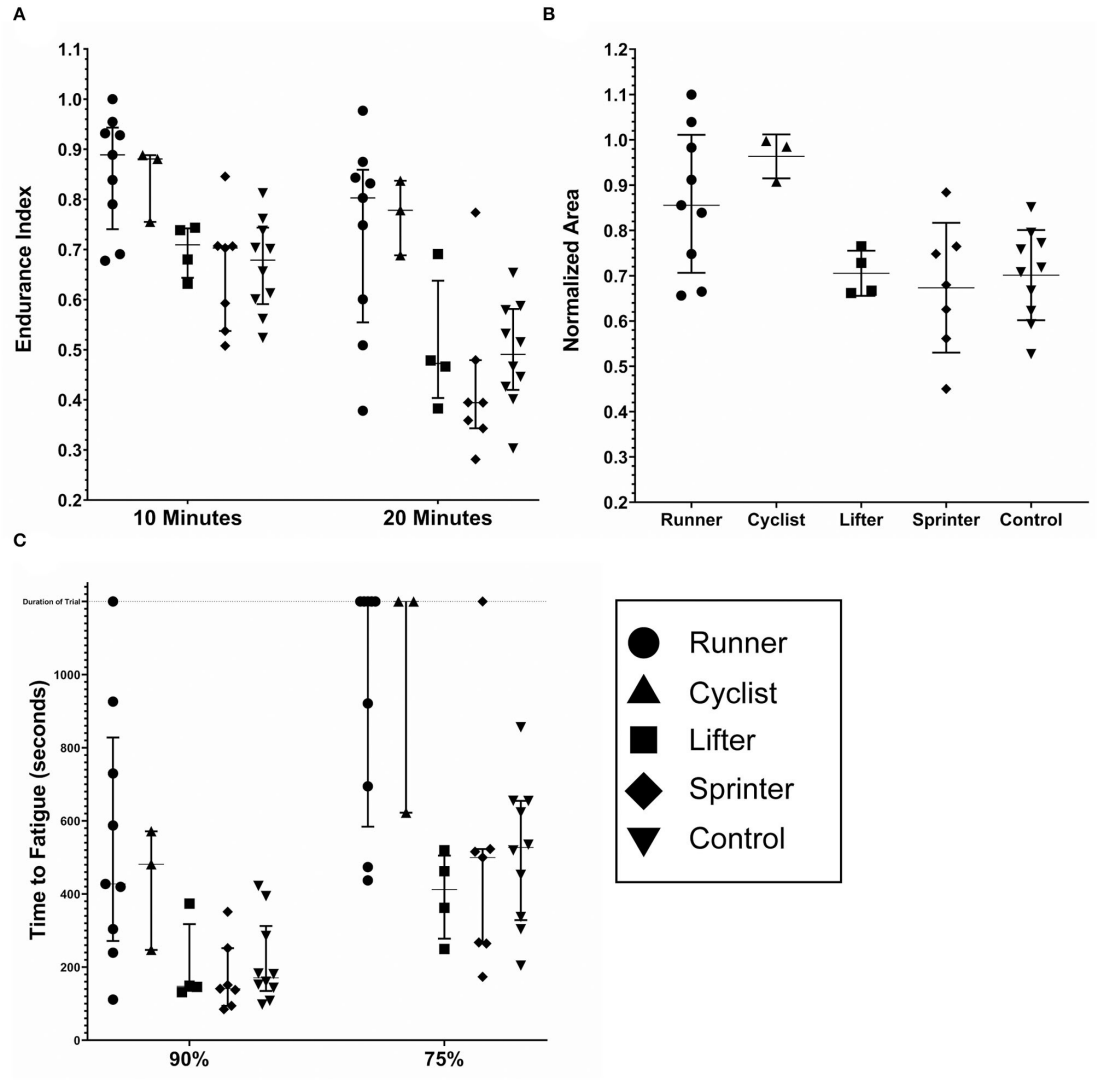
Fatigue metrics by sport; runners (*n* = 9), cyclists (*n* = 3), lifters (*n* = 4) sprinters (*n* = 7) and controls (*n* = 10). **(A)** The endurance index as measured at the 10 and 20 min timestamp. **(B)** The normalized area. Some subjects had calculated values above 1.0, due to the processing method. **(C)** The time to Fatigue to 90% and 75% of the initial force output. The duration of the trail was 1,200 s and is noted by the dashed horizontal line. The lines within each data set denote the median and the interquartile range.

The individual fatigue metrics when broken down by sport are shown in Figure 5: Runners (*n* = 9), Cyclists (*n* = 3), Lifters (*n* = 4), Sprinters (*n* = 7), Control (*n* = 10). The intended observation for this breakdown was any significance between sports within the same cohort (Runners vs. Cyclist, Lifter vs. Sprinter). In every metric, no significant difference between the sports within cohorts was observed. However, the low subject counts result in this analysis being underpowered, reducing the confidence of this observation. The observed ceiling effect in the TF metrics with the low subject count further reduced the confidence. Results of all three metrics between the five sports are summarized in Table 1.

**Table 1:** Mean (±SD) values of endurance indices (EI), normalized force area (NA), and time to fatigue (TF) metrics for sport.

	**Runner**	**Cyclist**	**Lifter**	**Sprinter**	**Control**
*n*	9	3	4	7	10
EI10	0.86 ± 0.11	0.84 ± 0.07	0.70 ± 0.05	0.66 ± 0.12	0.67 ± 0.1
EI20	0.73 ± 0.19	0.77 ± 0.07	0.5 ± 0.13	0.43 ± 0.16	0.49 ± 0.1
NA	0.87 ± 0.16	0.96 ± 0.05	0.71 ± 0.05	0.67 ± 0.14	0.70 ± 0.1
TF90 (seconds)	549 ± 349	443 ± 167	200 ± 116	173 ± 96	213 ± 115
TF75 (seconds)	947 ± 329	1,007 ± 333	398 ± 118	492 ± 343	514 ± 196

#### Gender

**Figure 6:**
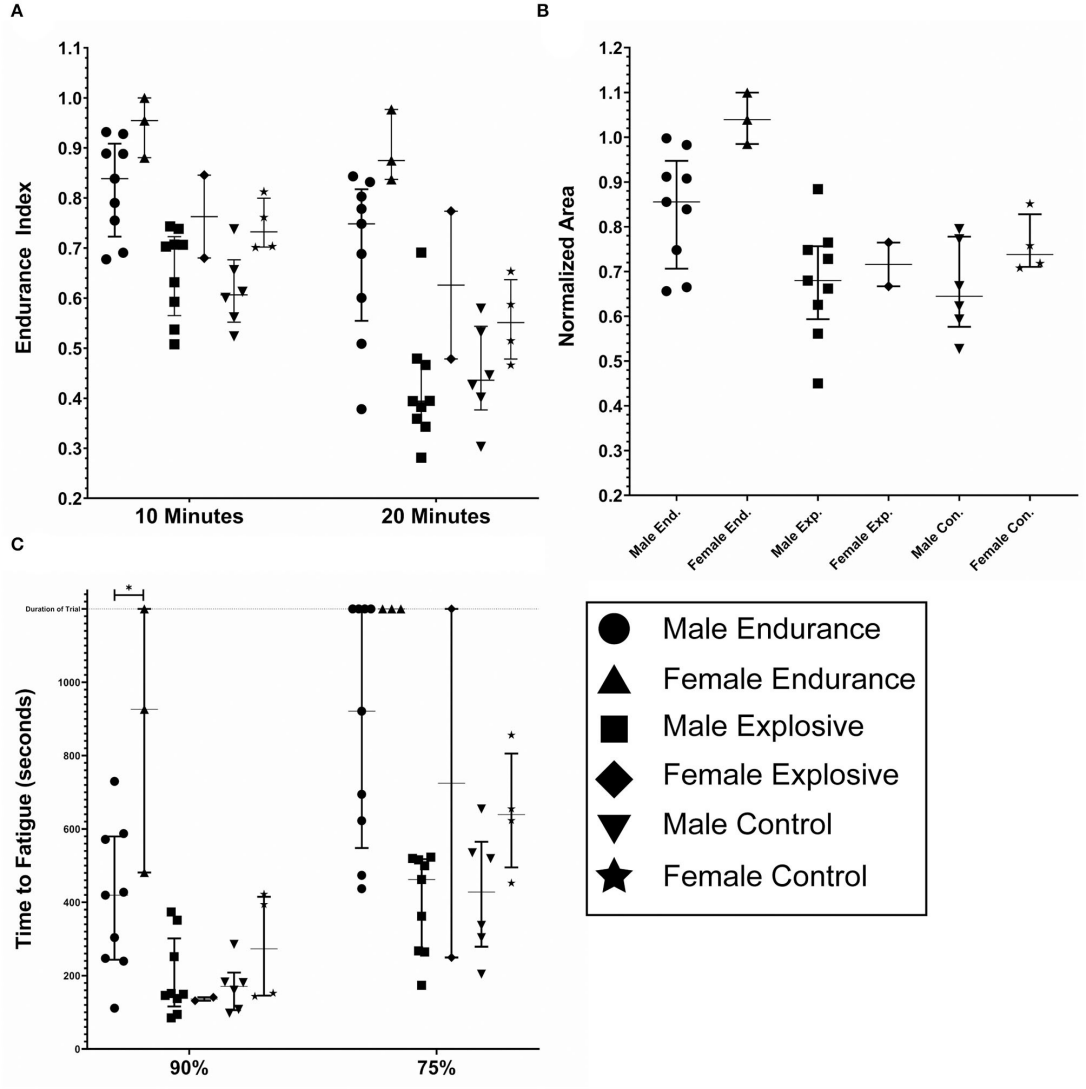
Fatigue metrics by gender; male endurance (*n* = 9), female endurance (*n* = 3), male explosive (*n* = 9), female explosive (*n* = 2), male control (*n* = 6), and female controls (*n* = 4). **(A)** The endurance index as measured at the 10 and 20 min timestamp. **(B)** The normalized area. Some subjects had calculated values above 1.0, due to the processing method. **(C)** The time to Fatigue to 90 and 75% of the initial force output. The duration of the trail was 1,200 seconds and is noted by the dashed horizontal line. The lines within each data set denote the median and the interquartile range.

Figure 6 depicts the fatigue metrics when broken down by gender within each Cohort: Male Endurance (*n* = 9), Female Endurance (*n* = 3), Male Explosive (*n* = 9), Female Explosive (*n* = 2), Male Control (*n* = 6), Female Control (*n* = 4). The intended observation for this breakdown was any significance between gender within the same cohort (i.e. Male vs. Female Endurance. In all metrics but TF90, where the Female Endurance fatigued significantly less than the Male Endurance (*p* < 0.05), there was no significance. There was however an observed trend that the females fatigued less than the males in all comparisons but explosive TF90. For example, all three female endurance athletes never fatigued below 75%. As was the case with the sport-specific breakdown, this analysis was underpowered and contained a ceiling effect so any trend, significant or otherwise, cannot be stated with confidence. A summary of the six gender groups is displayed in Table 2.

**Table 2:** Mean (±SD) values of endurance indices (EI), normalized force area (NA), and time to fatigue (TF) metrics for sport.

	**Male endurance**	**Female endurance**	**Male explosive**	**Female explosive**	**Male control**	**Female control**
*n*	9	3	9	2	6	4
EI10	0.82 ± 0.1	0.94 ± 0.06	0.65 ± 0.09	0.76 ± 0.12	0.62 ± 0.08	0.74 ± 0.05
EI20	0.69 ± 0.16	0.90 ± 0.07	0.42 ± 0.12	0.63 ± 0.20	0.45 ± 0.10	0.56 ± 0.08
NA	0.84 ± 0.13	1.04 ± 0.05	0.68 ± 0.13	0.72 ± 0.07	0.66 ± 0.10	0.76 ± 0.07
TF90 (seconds)	404 ± 199	869 ± 362	193 ± 107	136 ± 7	169 ± 67	278 ± 151
TF75 (seconds)	883 ± 330	1200 ± 0	399 ± 135	724 ± 672	426 ± 170	646 ± 165

## Discussion

The purpose of our study was to observe how differences in an individual's training type, and therefore muscle fiber type composition (Astrand et al., 1970; Costill et al., 1976; Tesch and Karlsson, 1985) or muscle enzyme composition (Foster et al., 1978; Hawley and Stepto, 2001), could affect the fatigability of muscle during repetitive NMES contractions. In each comparative metric, the endurance group fatigued significantly less than either the explosive group or the control group. While the explosive athletes were highly competitive individuals, they fatigued at a rate comparable to the non-trained controls. Therefore, when designing a NMES regimen for athletic training, the training of the specific athlete should be taken into account. For example, an endurance athlete can likely withstand a significantly greater number of repetitions or cadence in a strength training regimen. In recovery regimens, where fatigue is to be avoided, there may need to be fewer repetitions for an explosive athlete. Astronauts do not have a mandated preflight exercise regimen (Hackney et al., 2015), and an individual's personal training preference could therefore dictate how long of a NMES bout an individual could receive as a musculoskeletal countermeasure. Additionally, in spinal cord injury patients, who have significantly more Type II muscle fiber with little ability to perform endurance exercise, we would expect rapid fatigue like those of the explosive or control subjects. Therefore, when making a regimen that requires minimal fatigue, such as one that targets bone, the sets should be kept short. For example, with the particular duty cycle used in this study, bouts should be no longer than 3 to 5 min.

Individuals will have a varying distribution of motor end plates (Saitou et al., 2000) and therefore a varying sensitivity to a NMES pulse of a certain strength. The lack of significance between the achieved MVIC_VM_ and voltage acquired during calibration shows that some subjects did not require excessively high voltage to achieve the target MVIC_VM_, as could be required by a low number of accessible motor end plates, which would subsequently result in higher fatigue to the local motor end plates innervated. Body fat thickness will attenuate the electrical field created by the NMES pulse (Doheny et al., 2008), which in the case of our device, would require a higher voltage to deliver the necessary current. However, the lack of any correlation between the thigh body fat thickness, which was generally lower in the endurance group, and the voltage required show that the variations in body fat thickness superior to the VM did not influence the results. Last, the lack of any correlation between the quadriceps cross sectional area with that of the achieved MVIC_VM_ show that the size of the thigh, despite a standard electrode size across all subjects, did not influence the strength of the NMES pulse required, and therefore fatigue.

Our results imply that only high-capacity endurance training can reduce fatigue during standard NMES. Highly competitive endurance athletes tend to have an increased presence of slow twitch muscle fiber (Costill et al., 1976; Fink et al., 1977) as well as greater vascularization and mitochondria counts (Foster et al., 1978; Hawley and Stepto, 2001), allowing for greater oxidative capacity and ATP production (Holloszy and Coyle, 1984) in all muscle fibers (Henriksson, 1992). The presence of the outlier in the explosive group supports these interpretations. We recruited subjects based on their history and performance within a particular sport but did not inquire about additional training outside of their team or club. Follow up investigation revealed that the low fatiguing explosive athlete was training for a marathon outside of their competitive 400 m sprinting.

Our results were surprising due to the nature of the NMES contractions. NMES contractions recruit motor neurons and subsequent muscle fibers in the vicinity of the electric field (Bickel et al., 2011), and the contractions are unable to be modulated by the recruitment of additional muscle fibers or by increasing the signal frequency (Chou et al., 2008; Vromans and Faghri, 2017), resulting in rapid fatigue. Additionally, NMES contractions result in rapid glycogen depletion when compared to voluntary actions (Johnson et al., 2003). As fast twitch muscle fiber generates greater forces than slow twitch, it was thought that the fast twitch muscle would generate the majority of the force output in the MVICs and the NMES contractions. Combined with the tendency for fast twitch muscle to be more superficial in the quadriceps (Lexell et al., 1983), it was initially expected that all subjects would experience initial rapid fatigue, regardless of their own muscle fiber type composition, because the fatiguing of any amount of fast twitch muscle fiber would result in large force reductions relative to their own MVIC. This expected fatigue profile was previously observed in a prior study using relatively rapid repetitive NMES contractions to the VM (Lieber and Kelly, 1993).

Instead, the endurance athletes showed a surprising resilience to repetitive NMES contractions over a 20-min period. The lack of fatigue in the Endurance Indices showed a resistance to fatigue overall, and the lack of fatigue in the NA showed a consistent contractile pattern over the 20 min. While the fatigue profile observed varied between subjects (some linear, some the expected), the lack of fatigue in the Time to Fatigue showed that even if present, any initial rapid fatigue was less significant when compared to the explosive and control cohorts. While the exact reasoning cannot be determined due to the lack of muscle biopsies to determine muscle fiber type composition and enzyme levels, it can be assumed that these factors played a major role. It is possible that all the endurance athletes had such high slow twitch percent muscle composition, that most of their MVICs and NMES force output were from slow twitch and thus the force output was little affected relative to the MVIC. Conversely, it is possible that the extended activity increased the aerobic and oxidative capacity of the fast twitch muscle to a degree that it could better resist fatigue during NMES.

This study also included specific breakdowns by sport and gender within each cohort but the low subject counts in each category prevented sufficient power required to confidently note any difference, even in the situations where significance was achieved. First, there was no difference observed between the runners and the cyclists, implying that long duration aerobic activity, regardless of modality, can potentially reduce fatigability. The lack of any difference between the lifters and sprinters, activities that vary significantly in the magnitude of duration (1–2 vs. 20–50 s), also implies that only long duration aerobic activity may have any observable effect on fatigability and that the reduction of the fatigue may not scale evenly with the duration of activity. However, this observation would require a more comprehensive follow-up study, as this study did not include any shorter distance athletes, such 5 k or 10 k racers, who would fill the gap between a 400 m and half marathon. Second, while not significant, there was a tendency for the females of each cohort to fatigue less than the males. While the mechanisms are still not fully understood, prior studies have shown that female muscles tend to have a tendency to be more fatigue resistant and recover quicker when compared to male muscles in explosive and resistance type exercises (Fulco et al., 1999; Glenmark et al., 2004). The results observed here further confirm that tendency. Again, the low subject count, specifically females, calls for a follow on the study with a markedly increased subject count to confirm this observation.

### Limitations

We recruited competitive athletes from endurance and explosive sports to represent the extremes of muscle fiber type composition (Costill et al., 1976; Tesch and Karlsson, 1985), but as we were unable to obtain muscle biopsies or other noninvasive muscle fiber type determinations (Baguet et al., 2011), it cannot be said for certain whether the muscle characteristics of any individual matched their expected fiber type composition or the expected spatial distribution of the quadriceps (Lexell et al., 1983). Additionally, without a biopsy, we could not measure the concentration of specific mitochondrial enzymes, which can determine muscle functional characteristics such as oxidative capacity (Maltais et al., 2000). The presence of the higher fatiguing endurance athletes and lower fatiguing explosive athletes highlights these unknowns.

While the statistical significance of low *p* values was achieved, the inherent noise of the external force measurements should be considered. Rather than measuring torque at the knee joint, we measured external force at the shin. This increased the likelihood of erroneous peaks from voluntary movement, as well as an erroneous envelope during the “rest” if contact was reduced or lost, affecting numerous data points. This contributes to a potential lack of consistency in the fidelity of the data between subjects. Additionally, because of the noise inherent in our methods, we cannot say for certain whether the fatigue profiles observed in any subject were the true fatigue profiles and any analysis into investigating why some subjects apparently had the expected fatigue pattern and others did was not possible. Therefore, further studies should utilize a knee torque dynamometer if possible.

This study utilized a constant voltage, rather than a constant current stimulator. Therefore, differences in subject skin characteristics could affect the current delivered, and differences such as subject hair could affect electrode contact which could further alter the current delivered. However, as each subject was compared against themselves, these confounding factors were reduced. Additionally, the parameters of an NMES protocol, including the pulse characteristics and duty cycle, can have major effects on the fatiguability of a muscle during repeated contractions, and therefore the fatigue trends between the cohorts observed in our studies may differ from other NMES parameters. Repeating this study with different duty cycles, to include longer times would be beneficial.

Due to an inability to recruit athletes from large institutions such as Division 1 collegiate sports, there was a reduced number of subjects as well as an inherent variability in subject training even from within the same sport. If we could recruit athletes from collegiate teams, numerous subjects would have nearly identical training patterns, further reducing any potential variables. Instead, we recruited from the local community club teams, and while standards of competitive performance were dictated, we were unable to achieve the same confidence of training consistency between subjects of the same cohort and sport.

## Conclusion

Our study served as an initial investigation into what aspects of a healthy individual's muscle can influence fatigue and force output during NMES and looked at athletes of two extreme training modalities: endurance and explosive. Our results showed with high significance that only high capacity endurance training can reduce fatigue. High capacity explosive training had no effect on fatigue, with results comparable to an individual with minimal exercise frequency. While the limitations of this study prevent a clear physiological reason for why this occurs, the knowledge gained here can be used to aid in the development of NMES protocols for use in healthy populations such as for athletic performance, astronaut musculoskeletal countermeasures, as well as with spinal cord injury patients where fatigue is to be minimized.

## Data Availability Statement

The raw data supporting the conclusions of this article will be made available by the authors, without undue reservation.

## Ethics Statement

The studies involving human participants were reviewed and approved by Committee on the Use of Humans as Experimental Subjects (COUHES) Massachusetts Institute of Technology. The patients/participants provided their written informed consent to participate in this study.

## Author Contributions

TA conducted data collection, data analysis, and wrote the first draft of the manuscript. SR provided revisions. KD and DN read and approved final drafts of the manuscript. All authors contributed to the conception of the study and to the experimental design.

## Funding

This research was supported by the Charles Stark Draper Laboratory, Inc., Draper Scholar Program.

## Conflict of Interest

The authors declare that the research was conducted in the absence of any commercial or financial relationships that could be construed as a potential conflict of interest.

## Publisher's Note

All claims expressed in this article are solely those of the authors and do not necessarily represent those of their affiliated organizations, or those of the publisher, the editors and the reviewers. Any product that may be evaluated in this article, or claim that may be made by its manufacturer, is not guaranteed or endorsed by the publisher.
