# Supplementary material for: Effect of Athletic Training on Fatigue During Neuromuscular Electrical Stimulation
Source: Front Sports Act Living. 2022 Jun 14;4:894395. doi: 10.3389/fspor.2022.894395 (PMC9237484; doi:10.3389/fspor.2022.894395)
Supplement: Supplementary file 1 [file Data_Sheet_1.docx]

Table A 1: Individual Subject Information

|  | Sex | Age | Primary Sport/Event | Secondary (If noted) |
| --- | --- | --- | --- | --- |
| Endurance |  |  |  |  |
| 1 | M | 19 | Half Marathon |  |
| 2 | M | 25 | Half Marathon |  |
| 3 | M | 23 | Half Marathon |  |
| 4 | M | 28 | Marathon | Triathlon |
| 5 | M | 32 | Cyclist | Triathlon |
| 6 | F | 29 | Cyclist |  |
| 7 | F | 46 | Marathon | Triathlon |
| 8 | M | 22 | Marathon |  |
| 9^1^ | F | 32 | Marathon |  |
| 10 | M | 23 | Marathon |  |
| 11 | M | 32 | Cyclist |  |
| 12 | M | 19 | Half Marathon |  |
| 13 | F | 19 | Marathon |  |
| Explosive |  |  |  |  |
| 1 | M | 34 | Powerlifting | Strongman |
| 2 | M | 26 | Sprints |  |
| 3 | M | 43 | Powerlifting |  |
| 4 | M | 20 | Sprints |  |
| 5 | F | 34 | Olympic Weightlifting |  |
| 6 | F | 28 | Sprints |  |
| 7 | M | 37 | Sprints | High Jump |
| 8 | M | 22 | Hurdles | Sprints |
| 9 | M | 31 | Sprints | Powerlifting |
| 10 | M | 29 | Olympic Weightlifting |  |
| 11 | M | 31 | Sprints |  |
| Control |  |  |  |  |
| 1 | M | 32 |  |  |
| 2 | M | 24 |  |  |
| 3 | F | 19 |  |  |
| 4 | F | 23 |  |  |
| 5 | M | 24 |  |  |
| 6 | F | 33 |  |  |
| 7 | M | 23 |  |  |
| 8 | M | 26 |  |  |
| 9 | F | 27 |  |  |
| 10 | M | 28 |  |  |
| ^1^ Subject data excluded following completion of test. | | | | |

Table A 2: Subject Recruitment Inclusion Criteria by Sport

| **Sport** | **Inclusion Criteria** |
| --- | --- |
| Weightlifting | Candidate for Master of Sport (CMS) in weight class^1^ |
| Powerlifting | Class 1 in either the Squat or the Deadlift in weight class^2^ |
| Sprints | 400m or shorter; Active Participation on a competitive team for ≥ 5 years^3^ |
| Distance Running | Top 10% finish in event in past 12 months in Half Marathon or Above^4^ |
| Cyclist | At least one top 5% finish in a race in the past 12 months^5^ |
| ^1^ Adjusted Soviet Weightlifting Class System  ^2^ United States Powerlifting Association Classifications  ^3^ No comprehensive collection of performance percentiles.  ^4^ Marastat.com  ^5^ Road-Results.com | |

Table A 3: List of Subjects with Dynamometer Disconnections

| **Subject** | **Number of Missed Points** | **Time of Gap (seconds)^1^** |
| --- | --- | --- |
| Endurance 13 | 25 | 526 – 632 |
| Control 3 | 30 | 460 – 588 |
| Control 4 | 14 | 524 – 584 |
| Control 5 | 13 | 124 – 184 |
| Control 6 | 17 | 512 – 586 |
| Control 7 | 19 | 506 – 584 |
| Control 8 | 25 | 376 – 482 |
| Control 9 | 15 | 504 – 574 |
| Control 10 | 27 | 220 - 332 |
| ^1^ Test time-lapse was 0 to 1200 seconds | |  |
